# Detection of ERG11 gene mutation in coding and non-coding regions of clinical Candida glabrata (Nakaseomyces glabratus) isolates from Pakistan

**DOI:** 10.1099/acmi.0.000952.v6

**Published:** 2025-09-19

**Authors:** Saba Memon, Najia Karim Ghanchi, Urooj Zafar, Joveria Farooqi, Sadaf Zaka, Kauser Jabeen

**Affiliations:** 1 Department of Pathology & Laboratory Medicine, Aga Khan University, Karachi, Pakistan; 2 Department of Microbiology, University of Karachi, Karachi, Pakistan

**Keywords:** c. -66 T/G, *Candida glabrata*, *ERG11 *gene, molecular resistance, *Nakaseomyces glabratus*, Pakistan

## Abstract

Azoles inhibit the cytochrome P450-dependent enzyme lanosterol 14α-demethylase (*CYP51*) that is encoded by the *ERG11* gene. Azole resistance in *Candida* species arises through different mechanisms, like mutations in the *ERG11* gene, *ERG11* overexpression, CDR1,2 (*Candida* drug resistance) overexpression that actively efflux azole drugs, reducing their intracellular concentration and therapeutic effectiveness, and biofilm formation. We sequenced the *ERG11* gene to determine mutations in the coding and non-coding regions of *ERG11* in clinical isolates of *Candida glabrata* (*Nakaseomyces glabratus*) from Pakistan. Eight *C. glabrata* (*N. glabratus*) strains from our fungal strain bank (five fluconazole-resistant and three susceptible dose-dependent) were revived and used. The *ERG11* gene was amplified by PCR, sequenced using the Sanger methodology and analysed using bioinformatic tools. We identified a change in nucleotide at c. -66 T/G upstream of the start codon ATG in the promoter region of the *ERG11* gene in fluconazole-resistant *C. glabrata* (*N. glabratus*). Within the downstream (coding region), where numbering begins at the ATG start codon as position +1, two novel synonymous mutations at positions T300C and T834C and previously reported synonymous mutations T768C, A1023G, T1557A and A1581G were also observed. This is the first study evaluating *ERG11* mutations in *C. glabrata* (*N. glabratus*) from Pakistan. The clinical significance of such uncommon *ERG11* gene mutations, such as c. -66 T/G, should be explored further through correlation with treatment outcome data.

## Data Summary

All data are presented in the manuscript and supplementary materials. DNA sequences obtained throughout the work have been made publicly available in GenBank (PQ160094–PQ160100 and PQ047143–PQ047145).

## Introduction

*Candida glabrata* (*Nakaseomyces glabratus*) has been included in the recently published World Health Organization (WHO) fungal priority pathogens list to guide research, development and public health action [1]. *C. glabrata* (*N. glabratus*) exhibits reduced susceptibility to azoles, which are the most common agents prescribed for the management of candidiasis. This species also has the potential to rapidly develop resistance in response to exposure to azoles or echinocandins [2]. Azoles disrupt the ergosterol biosynthesis pathway for fungal cell membranes. There are around 20 enzymes in the cytochrome P450 family (CYP51) involved in the biosynthesis of ergosterol, of which 14α-demethylase encoded by *ERG11* is the key enzyme [3,4]. Mutations in the *ERG11* gene that result in amino acid changes in the enzyme sequence represent a major mechanism conferring resistance to azoles in *Candida* spp*.* [5].

In Pakistan, *C. glabrata* (*N. glabratus*) is a common agent of invasive candidiasis, particularly in adults [6]. In our previous study, we found a high rate of fluconazole resistance (18.4% of 261 strains), with fluconazole MICs of resistance strains ranging from 64 to 256 mg l^−1^ [7]. Although azole resistance has emerged in *C. glabrata* (*N. glabratus*) in Pakistan, data on molecular determinants of azole resistance are not available. This study reports the presence of mutations in the coding and non-coding regions of the *ERG11* gene of clinical *C. glabrata* (*N. glabratus*) isolates.

## Methods

### Study site and setting

This observational study was conducted from 2019 to 2020 at the Aga Khan University (AKU) clinical laboratories, Karachi, Pakistan. The study was approved by the AKU Ethics Review Committee (AKU-ERC number: 2019-0438-2659).

### Phenotypic identification of strains

Eight invasive *C. glabrata* (*N. glabratus*) isolates [five fluconazole-resistant and three susceptible dose-dependent (SDD)] were retrieved from a strain collection saved from a previous study [7] and analysed for *ERG11* gene mutation. Isolates were saved from pure culture by transferring to 50% glycerol phosphate broth and banked at −80 °C. Banked isolates were re-cultured on potato dextrose agar and incubated at 37 °C for 48 h. Growth of colonies was confirmed through standard phenotypic identification protocols. For this purpose, BBL™ CHROMagar™ Candida (BD, USA), Bismuth Glucose Glycine Yeast Agar (Oxoid, UK) and Corn Meal Agar with Tween 80 (CMT; Oxoid, UK) were used. On CHROMagar, a characteristic pink colour was observed. On Bismuth Glucose Glycine Yeast Agar (BiGGY), yeast colonies appeared as dome-shaped, light brown to white colonies, 2–3 mm in diameter in size, and on CMT, the Dalmau method showed tiny oval budding yeast cells without pseudohyphae.

### Biochemical identification of strains

API 20C Aux (bioMérieux, France) was used to confirm the biochemical profile. Manufacturer’s instructions for inoculating, incubating and interpreting the assimilation reactions for different types of sugars were followed. The results were interpreted according to the bioMérieux Analytic Profile Index database for *Candida*, available online [8]. Identification was confirmed as *C. glabrata* (*N. glabratus*) if the match was termed Acceptable, Good, Very Good or Excellent by the database.

### Antifungal susceptibility by MICs

MICs were determined using colorimetric broth microdilution with Roswell Park Memorial Institute 1640 broth (Sigma-Aldrich, UK), using the YeastOne Sensititre plates (Trek Diagnostics System Ltd., East Grinstead, England) [7], and interpreted according to Clinical Laboratory Standards Institute breakpoints mentioned in ‘Performance standard for antifungal susceptibility testing of yeasts’ (M60-ED: 2017) [9]. The susceptibility of *C. glabrata* (*N. glabratus*) isolates was interpreted as follows: isolates with fluconazole MIC ≤32 mg l^−1^ were categorized as SDD, while those with MIC ≥64 mg l^−1^ were considered resistant.

### Characteristics of study strains

The selected strains had fluconazole MICs ranging from 2 to 256 mg l^−1^ [7]. Three isolates were from different hospitals in Karachi (two fluconazole-resistant and one SDD), one isolate from other cities in Sindh, one isolate from Punjab (each fluconazole-resistant) and three isolates were taken from AKU inpatients (one fluconazole-resistant and two SDD).

### DNA extraction

Yeast colonies grown for 24 h on sabouraud dextrose agar with chloramphenicol (SDAC) medium were homogenized at 4,000 r.p.m. for three cycles of 90 s=0.05 using Precellys 24 lysis (Bertin Technologies). Genomic DNA was subsequently extracted following the manufacturer’s protocol provided with the Qiagen mini kit (QIAquick, Germany). DNA concentration and purity were determined using a NanoDrop (DeNovix DS-11 FX +Spectrophotometer/Fluorometer).

### Molecular identification

The molecular identification of strains was achieved by sequencing of internal transcribed spacer (ITS) region. The universal primers ITS1 (5′-TCC GTA GGT GAA CCT GCG G-3′) and ITS4 (5′-TCC TCC GCT TAT TGA TAT GC-3′) were used under cyclic conditions that were previously described [10].

### Amplification of *ERG11* gene

The *ERG11* gene was amplified using primers and conditions previously described for *C. glabrata* (*N. glabratus*) [3]. Briefly, amplification reactions were performed in a 25 µl reaction volume containing 12.5 µl master mix (HotStar master mix), 1 µl each of forward and reverse primers (10 pmol µl^−1^) and 2 µl of 25 ng genomic DNA. The reaction conditions involved initial denaturation at 94 °C for 1 min, 30 denaturation cycles at 94 °C for 30 s, annealing at 50 °C for 40 s, extension at 72 °C for 50 s, followed by a final extension at 72 °C for 10 min. The PCR products were analysed on 1.5% agarose gels. Amplified products were purified using a PCR purification kit (QIAquick, Germany). Purified products were shipped to Eurofins Genomics for complete Sanger sequencing using sequencing primers previously described [3].

### Sequence analysis

**Fig. 1.**
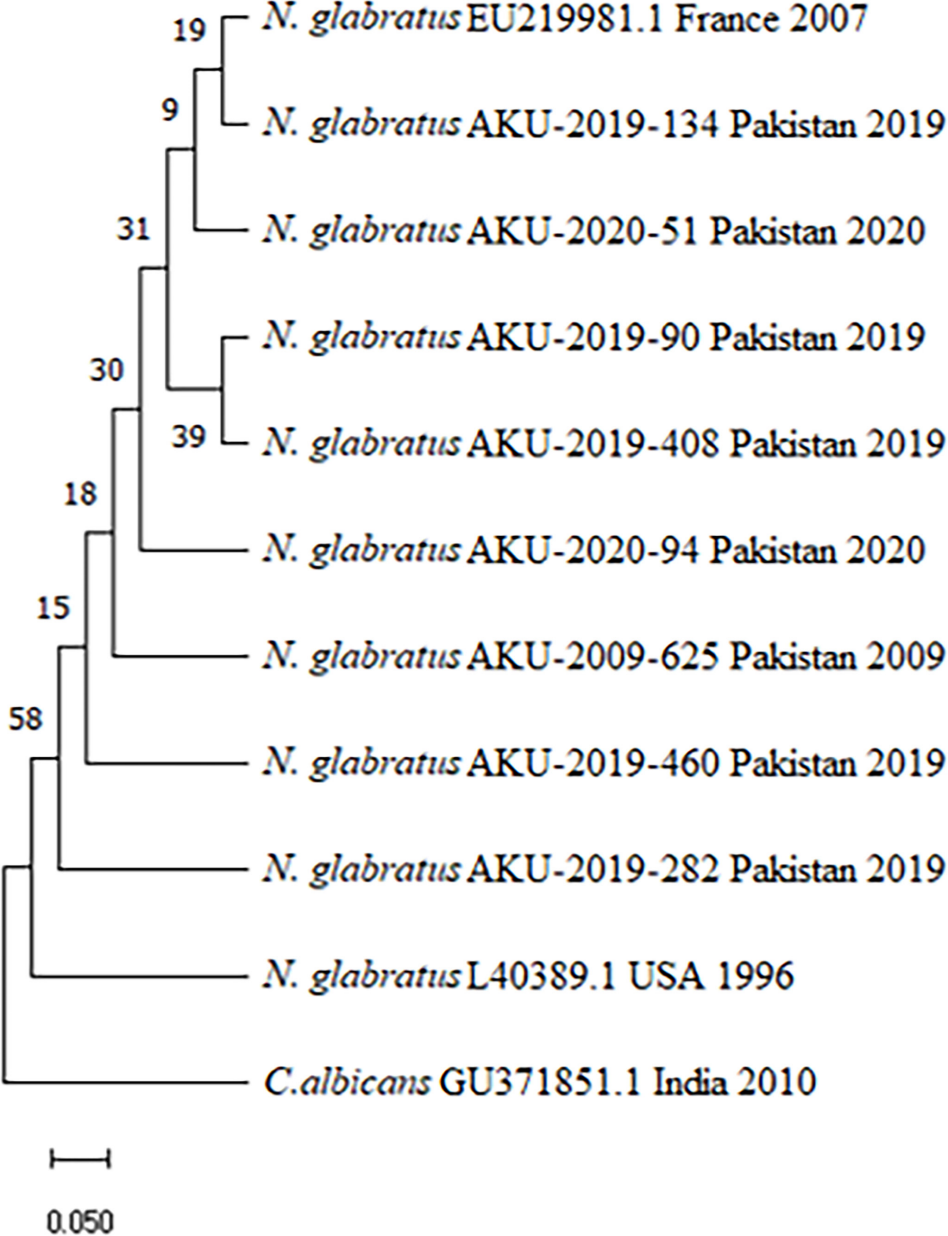
Phylogeny of the *ERG11* gene of *N. glabratus* and reference sequence. A neighbour-joining tree was computed using the Tajima-Nei method [22]. Bootstrap values (>50%) from 1,000 replicates are displayed at the branches; the tree depicts limited genetic variation among closely related isolates, with a scale bar indicating branch length. The analysis included 11 sequences: eight *N. glabratus* isolates with year and origin and two reference sequences of *N. glabratus –* EU219981.1 is used to group and identify related strains and L40389.1 provides contrast and evolutionary distance. The *C. albicans* GU371851.1 serves as an outgroup [23,24]. Nodes (where branches split) represent common ancestors. The deeper the node, the older the shared ancestor.

A consensus *ERG11* gene complete sequence of *C. glabrata* (*N. glabratus*) was established using the CAP3 software (https://www.insilico.uni-duesseldorf.de/Cap3.html). Data were entered for alignment in Molecular Evolutionary Genetics Analysis (mega 11) using ClustalW (pairwise and multiple alignment) with previously published reference sequences and L40389.1 from France (2007) and Washington, D.C., USA (1996), respectively [3,11] (Fig. 1). The ITS and *ERG11* sequences (three ITS sequences: PQ047143, PQ047144 and PQ047145; and eight *ERG11* sequences: PQ160100, PQ160097, PQ160094, PQ160098, PQ160095, PQ160096, PQ160099 and PV867500) were deposited in the National Center for Biotechnology Information (NCBI) database. The consensus sequences were translated into their corresponding amino acid sequences using the ExPASy Translate Tool (https://web.expasy.org/translate/). BioEdit Sequence Alignment Editor was used for positions of amino acid and protein alignments to locate non-synonymous and synonymous mutations. A phylogenetic tree of *ERG11* sequences of *C. glabrata* (*N. glabratus*) isolates was constructed using the neighbour-joining method by using mega 11.

## Results

The phenotypic identification of *N. glabratus* on different media revealed distinct features. On CHROMagar, a characteristic pink colour was observed. On BiGGY, yeast colonies appeared as dome-shaped, light brown to white colonies, 2–3 mm in diameter in size, and on CMT, the Dalmau method showed tiny oval budding yeast cells without pseudohyphae.

The *ERG11* gene of eight isolates of *N. glabratus* was analysed in this study. Upon sequencing, sequences of 950 bp were obtained and showed 90% similarity with other strains of *N. glabratus* deposited in GenBank (Fig. S1, available in the online Supplementary Material). The median (range) MIC for fluconazole was 128 (2–256) mg l^−1^. Sequencing results of the coding and non-coding regions of *ERG11* from these isolates are shown in Table 1. Four strains (AKU-2020-51, AKU-2020-94, AKU-2019-90 and AKU-2019-282) exhibited cross-resistance with other triazoles, and two strains (AKU-2009-625 and AKU-2019-460) showed isolated non-WT MICs against voriconazole. Amphotericin MIC was within the susceptible range in all isolates.

**Table 1.** Distribution of MICs and *ERG11* non-synonymous and synonymous mutations in studied *N. glabratus* clinical isolates from Pakistan

Strain no.	Sample/accession no.	Location	Source	MIC (mg l^−1^)					*ERG11* mutations
				FLU	VOR	POS	ITR	AMB	
1	AKU-2009-625/PQ160100	Karachi	Blood	8	***0.5***	1	0.5	0.25	T300C†, T768C
2	AKU-2019-90/PQ160097	Karachi	Abdominal pus	***128***	***4***	***8***	***16***	0.5	T768C, T834C†, A1023G
3	AKU-2019-134/PQ160094	Karachi	Blood	2	0.12	0.5	0.25	0.25	T300C†, T768C, T834C†, A1023G, T1557A, A1581G
4	AKU-2019-282/PQ160098	Sindh	Blood	***64***	***0.5***	1	1	0.5	T768C
5	AKU-2019-408* PV867500	Karachi	Urine collected from kidney	***128***	na	na	na	0.5	**c.-496T/G**, T768C, T834C†, A1023G
6	AKU-2019-460/PQ160095	Karachi	Blood	16	***0.5***	1	0.5	0.25	T768C, T1557A, A1581G
7	AKU-2020-51/PQ160096	Punjab	Abdominal pus	***64***	***8***	***8***	4	0.25	T300C*†,T768C, T834C*†,A1023G
8	AKU-2020-94/PQ160099	Karachi	Blood	***256***	***8***	***8***	***16***	0.5	T768C, A1023G

Bold: non-synonymous mutation, change in nucleotide upstream of the start codon ATG: **c.-496T/G** (base substitution) in promoter region of *ERG11 *gene*. Bold and italicized: resistant or non-WT MIC.

†Novel synonymous mutation.

AMB, amphotericin; FLU, fluconazole; ITR, itraconazole; na, not available; POS, posaconazole; VOR, voriconazole.

We identified a change in nucleotide at c. -66 T/G upstream of the start codon ATG in the promoter region of the *ERG11* gene in fluconazole-resistant *C. glabrata* (*N. glabratus*), AKU-2019-408. Since mutations in the promoter region can alter transcription factor binding and subsequently affect gene expression, this upstream substitution may contribute to resistance by enhancing *ERG11* transcription. No non-synonymous mutations were detected within the downstream coding region, where nucleotide numbering begins at the ATG start codon as position +1, indicating that the protein sequence of lanosterol 14α-demethylase remained unchanged. Novel synonymous mutations at positions T300C and T834C were observed in 3/8 (38%) and 4/8 (50%) of the isolates, respectively (Fig. S1). Previously reported synonymous mutations T768C, A1023G, T1557A and A1581G in the *ERG11* gene were also observed in 8/8 (100%), 5/8 (63%) and 2/8 (25%) of the isolates, respectively (Table 1 and Fig. S1a, b).

The phylogenetic tree showed that all tested strains and reference sequences obtained from the database are rooted at the same point. Although they share a common ancestry, splitting of branches further in between studied isolates demonstrated the mutations. The reference strain from Washington, D.C., USA (L40389.1; 1996) showed greater similarity with five of our test strains except three, AKU-2019-282, AKU-2019-460 and AKU-2020-94. The similarity values between the eight tested isolates and the reference sequence EU219981 in the NCBI database were higher than 98%, with the highest similarity of 99.32% observed in sample ID-AKU-2019-134. The phylogenetic tree analysis of the *ERG11* gene of *N. glabratus* suggests the presence of that same clade in both AKU-2019-90 and AKU-2019-408, with the same phenotypic MICs and synonymous mutation. Isolate numbers AKU-2019-134 and 51 had different phenotypic MICs, but nucleotide sequence showed similar pattern of synonymous mutation (Table 1). AKU-2009-625 is from distant clades with low fluconazole MICs and one of three strains that showed novel synonymous mutation (Fig. 1).

## Discussion

The prevalence of higher azole resistance is attributed to mutations in the *ERG* gene pathway. Y132F and K143R *ERG11* substitutions are considered major causes of fluconazole resistance in *Candida albicans* and are also associated with triazole resistance in *Candidozyma auris*, *Candida parapsilosis* and *Candida tropicalis*, but not in *N. glabratus* [12,13]. *N. glabratus* is intrinsically less susceptible to azoles and easily develops resistance; however, limited evidence of mutations in the *ERG11* gene is available. Triazole resistance associated with *ERG11* frequently shows cross-resistance between fluconazole and voriconazole [13].

Single nucleotide polymorphisms associated with azole resistance in *N. glabratus ERG11* gene are reported at positions Y141H, N368T and L381M [14]. In our study, non-synonymous mutations were absent in the coding region of *N. glabratus* isolates, while a c. -66 T/G change in nucleotide was noted upstream of the start codon ATG in the non-coding region of the *ERG11* gene in one fluconazole-resistant isolate. All six strains showing resistance and non-WT MICs against other triazoles had a common synonymous mutation, T834C. However, the presence of C423T, T768C, A1023G, T1557A and A1581G mutations in both azole-resistant and -sensitive *N. glabratus* isolates implies that these changes might not play a role in conferring resistance to antifungal agents as observed in previous studies [3].

Similarly, novel synonymous mutations in the coding region, T300C and T834C, were found in our isolates. Mutations in coding region, T768C and A1023G, were predominant in our isolates at all ranges of fluconazole MICs (2–256 mg l^−1^), similar to findings by Vandeputte *et al*. [15] and Silva *et al*. [3], implying that these changes may not play a role in conferring resistance to azole antifungal agents. Although synonymous mutations do not change the amino acid sequence, studies have reported that these could play a role in destabilizing mRNA and facilitating its unfolding for efficient translation [16,18]. However, their significance remains to be established through transcriptional and translational studies [14,17]. It was found that *N. glabratus* has other mechanisms of resistance: exogenous (human) sterol uptake [2], effects on the expression of Pdr1/*CDR1* and, more rarely, mutations in the *ERG11* gene. The most common amino acid substitution mutations in *N. glabratus* are in the gene encoding the Zn2Cys6 called Pdr1.

The mutation c. -66 T/G change in nucleotide was noted upstream of the start codon ATG region of *ERG11* and has previously been reported, yet a clear association with resistance has not been demonstrated [14,19]. However, in our case, strain number 5 (AKU-2019-408) with c. -66 T/G mutation showed phenotypic resistance to fluconazole. Non-coding regions constitute a significant part of eukaryotic genomes; their roles in fungi are just starting to emerge. They participate in the regulation of gene expression in response to varying environmental conditions, fungal pathogenesis and antifungal drug response [20]. Strains carrying such *ERG11* gene mutations, which are unique to geographical regions, should be investigated through regional resistance and genotypic surveillance, as well as clinical response to treatment to help study the development of resistance [21].

The phylogenetic tree generated from the coding and non-coding region sequences of the *N. glabratus ERG11* gene indicates that AKU-2019-90 and AKU-2019-408 are in the same clade, having same phenotypic and genotypic values and highest similarity to reference sequence EU219981.1.

To our knowledge, this is the first report presenting the commonly reported and novel mutations in local invasive *N. glabratus* isolates from Pakistan. Further studies using a whole-genome sequencing-based approach might clarify the mechanism of reduced azole sensitivity observed, especially in resistant *N. glabratus*. A comparative genomic study of these clinical isolates with the local environmental isolates might suggest the mechanism of genome plasticity among these isolates. Nevertheless, the present study has limitations because it is a single-centre study with a very small proportion of isolates from outside Karachi and a small sample size. Additional studies to explore Pdr1/*CDR1* gene are needed to assess its role in conferring resistance to azoles in *N. glabratus* strains.

## Conclusion

This is the first study of *ERG11* mutations in *N. glabratus* species from Pakistan. Most of the tested strains did not have non-synonymous and synonymous polymorphisms. Only one of the strains had a mutation at codon c. -66. We propose genomic surveillance on a larger number of strains and correlation with clinical correlation to establish significance of this mutation.

## Supplementary material

10.1099/acmi.0.000952.v6 Uncited Fig. S1.

## Abbreviations

AKU: Aga Khan University
CMT: Corn Meal Agar with Tween 80
ITS: internal transcribed spacer
NCBI: National Center for Biotechnology Information
SDAC: sabouraud dextrose agar with chloramphenicol
