# Supplementary material for: Detection of ERG11 gene mutation in coding and non-coding regions of clinical Candida glabrata (Nakaseomyces glabratus) isolates from Pakistan
Source: Access Microbiol. 2025 Sep 19;7(9):000952.v6. doi: 10.1099/acmi.0.000952.v6 (PMC12451296; doi:10.1099/acmi.0.000952.v6)
Supplement: Uncited Fig. S1. [file acmi-7-00952-s001.pdf]

```
                    460        470        480        490        500        510        520        530        540
            ....|....| ....|....| ....|....| ....|....| ....|....| ....|....| ....|....| ....|....| ....|....|
L40389.1     AAAAATGTAT ATAATGGGCG ATCCCTTCAT GTCCATTGTC TTGAATTTCT TGTTCTCATT GTCTATTTCG ATACTACAAA CCACATAAAT
EU219981.1   .......... .......... .......... .......... .......... .......... .......... .......... ..........
AKU-2009-625 .......... .......... .......... .......... .......... .......... .......... .......... ..........
AKU-2019-90  .......... .......... .......... .......... .......... .......... .......... .......... ..........
AKU-2019-134 .......... .......... .......... .......... .......... .......... .......... .......... ..........
AKU-2019-282 .......... .......... .......... .......... .......... .......... .......... .......... ..........
AKU-2019-408 .......... .......... .......... .......... .....G.... .......... .......... .......... ..........
AKU-2019-460 .......... .......... .......... .......... .......... .......... .......... .......... ..........
AKU-2020-51  .......... .......... .......... .......... .......... .......... .......... .......... ..........
AKU-2020-94  .......... .......... .......... .......... .......... .......... .......... .......... ..........
```

Supplementary Figure 1 a: change in nucleotide upstream of start codon c. -66 T/G upstream (base substitution) of *ERG11* gene of *N. glabratus*. Accessions numbers L40389.1 and EU219981.1 were used as reference sequence. For alignment BioEdit software was used. Synonymous mutation at position c. -66 T/G in isolate number AKU- 2019-408. "*" represents not readable, "."represents similarity (no change of nucleotide).

```
                     10         20         30         40         50         60         70         80         90
             ....|....| ....|....| ....|....| ....|....| ....|....| ....|....| ....|....| ....|....| ....|....|
L40389.1     ATGTCCACTG AAAACACTTC TTTGGTCGTT GAACTATTGG AGTACGTGAA GCTTGGTCTT TCGTACTTCC AAGCTCTGCC ATTGGCGCAG
EU219981.1   .......... .......... .......... .......... .......... .......... .......... .......... ..........
AKU-2009-625 .......... .......... .......... .......... .......... .......... .......... .......... ..........
AKU-2019-90  .......... .......... .......... .......... .......... .......... .......... .......... ..........
AKU-2019-134 .......... .......... .......... .......... .......... .......... .......... .......... ..........
AKU-2019-282 .......... .......... .......... .......... .......... .......... .......... .......... ..........
AKU-2019-408 .......... .......... .......... .......... .......... .......... .......... .......... ..........
AKU-2019-460 .......... .......... .......... .......... .......... .......... .......... .......... ..........
AKU-2020-51  .......... .......... .......... .......... .......... .......... .......... .......... ..........
AKU-2020-94  .......... .......... .......... .......... .......... .......... .......... .......... ..........

                    100        110        120        130        140        150        160        170        180
             ....|....| ....|....| ....|....| ....|....| ....|....| ....|....| ....|....| ....|....| ....|....|
L40389.1     AGAGTGTCTA TTATGGTCGC CTTGCCATTT GTGTACACCA TCACATGGCA ATTGCTTTAC TCCTTGAGAA AGGACAGACC ACCACTTGTG
EU219981.1   .......... .......... .......... .......... .......... .......... .......... .......... ..........
AKU-2009-625 .......... .......... .......... .......... .......... .......... .......... .......... ..........
AKU-2019-90  .......... .......... .......... .......... .......... .......... .......... .......... ..........
AKU-2019-134 .......... .......... .......... .......... .......... .......... .......... .......... ..........
AKU-2019-282 .......... .......... .......... .......... .......... .......... .......... .......... ..........
AKU-2019-408 .......... .......... .......... .......... .......... .......... .......... .......... ..........
AKU-2019-460 .......... .......... .......... .......... .......... .......... .......... .......... ..........
AKU-2020-51  .......... .......... .......... .......... .......... .......... .......... .......... ..........
AKU-2020-94  .......... .......... .......... .......... .......... .......... .......... .......... ..........

                    190        200        210        220        230        240        250        260        270
             ....|....| ....|....| ....|....| ....|....| ....|....| ....|....| ....|....| ....|....| ....|....|
L40389.1     TTCTACTGGA TCCCATGGGT CGGCTCTGCT ATCCCATACG GTACCAAGCC ATACGAGTTC TTCGAAGACT GCCAAAAGAA ATACGGTGAT
EU219981.1   .......... .......... .......... .......... .......... .......... .......... .......... ..........
AKU-2009-625 .......... .......... .......... .......... .......... .......... .......... .......... ..........
AKU-2019-90  .......... .......... .......... .......... .......... .......... .......... .......... ..........
AKU-2019-134 .......... .......... .......... .......... .......... .......... .......... .......... ..........
AKU-2019-282 .......... .......... .......... .......... .......... .......... .......... .......... ..........
AKU-2019-408 .......... .......... .......... .......... .......... .......... .......... .......... ..........
AKU-2019-460 .......... .......... .......... .......... .......... .......... .......... .......... ..........
AKU-2020-51  .......... .......... .......... .......... .......... .......... .......... .......... ..........
AKU-2020-94  .......... .......... .......... .......... .......... .......... .......... .......... ..........

                    280        290        300        310        320        330        340        350        360
             ....|....| ....|....| ....|....| ....|....| ....|....| ....|....| ....|....| ....|....| ....|....|
L40389.1     ATCTTCTCTT TCATGCTATT GGGTAGAATT ATGACTGTCT ACTTGGGTCC AAAGGGTCAC GAATTCATCT TCAACGCCAA GTTGGCCGAT
EU219981.1   .......... .......... .......... .......... .......... .......... .......... .......... ..........
AKU-2009-625 .......... .......... .........C .......... .......... .......... .......... .......... ..........
AKU-2019-90  .......... .......... .......... .......... .......... .......... .......... .......... ..........
AKU-2019-134 .......... .......... .........C .......... .......... .......... .......... .......... ..........
AKU-2019-282 .......... .......... .......... .......... .......... .......... .......... .......... ..........
AKU-2019-408 .......... .......... .......... .......... .......... .......... .......... .......... ..........
AKU-2019-460 .......... .......... .......... .......... .......... .......... .......... .......... ..........
AKU-2020-51  .......... .......... .........C .......... .......... .......... .......... .......... ..........
AKU-2020-94  .......... .......... .......... .......... .......... .......... .......... .......... ..........

                    370        380        390        400        410        420        430        440        450
             ....|....| ....|....| ....|....| ....|....| ....|....| ....|....| ....|....| ....|....| ....|....|
L40389.1     GTTTCCGCTG AAGCTGCTTA CTCCCACTTG ACCACCCCAG TGTTCGGTAA AGGTGTTATC TACGATTGTC CAAACCACAG ACTAATGGAA
EU219981.1   .......... .......... .......... .......... .......... .......... .......... .......... ..........
AKU-2009-625 .......... .......... .......... .......... .......... .......... .......... .......... ..........
AKU-2019-90  .......... .......... .......... .......... .......... .......... .......... .......... ..........
AKU-2019-134 .......... .......... .......... .......... .......... .......... .......... .......... ..........
AKU-2019-282 .......... .......... .......... .......... .......... .......... .......... .......... ..........
AKU-2019-408 .......... .......... .......... .......... .......... .......... .......... .......... ..........
```

```
AKU-2019-460   .......... .......... .......... .......... .......... .......... .......... .......... ..........
AKU-2020-51    .......... .......... .......... .......... .......... .......... .......... .......... ..........
AKU-2020-94    .......... .......... .......... .......... .......... .......... .......... .......... ..........

                      460        470        480        490        500        510        520        530        540
               ....|....| ....|....| ....|....| ....|....| ....|....| ....|....| ....|....| ....|....| ....|....|
L40389.1       CAAAAGAAGT TTGTCAAGGG TGCTTTGACT AAGGAAGCCT TTGTCAGATA CGTTCCATTG ATCGCTGAGG AAATCTACAA GTACTTCAGA
EU219981.1     .......... .......... .......... .......... .......... .......... .......... .......... ..........
AKU-2009-625   .......... .......... .......... .......... .......... .......... .......... .......... ..........
AKU-2019-90    .......... .......... .......... .......... .......... .......... .......... .......... ..........
AKU-2019-134   .......... .......... .......... .......... .......... .......... .......... .......... ..........
AKU-2019-282   .......... .......... .......... .......... .......... .......... .......... .......... ..........
AKU-2019-408   .......... .......... .......... .......... .......... .......... .......... .......... ..........
AKU-2019-460   .......... .......... .......... .......... .......... .......... .......... .......... ..........
AKU-2020-51    .......... .......... .......... .......... .......... .......... .......... .......... ..........
AKU-2020-94    .......... .......... .......... .......... .......... .......... .......... .......... ..........

                      550        560        570        580        590        600        610        620        630
               ....|....| ....|....| ....|....| ....|....| ....|....| ....|....| ....|....| ....|....| ....|....|
L40389.1       AACTCCAAGA ACTTCAAGAT CAACGAAAAC AACTCCGGTA TCGTCGACGT TATGGTCTCC CAACCTGAAA TGACTATCTT CACTGCTTCC
EU219981.1     .......... .......... .......... .......... .......... .......... .......... .......... ..........
AKU-2009-625   .......... .......... .......... .......... .......... .......... .......... .......... ..........
AKU-2019-90    .......... .......... .......... .......... .......... .......... .......... .......... ..........
AKU-2019-134   .......... .......... .......... .......... .......... .......... .......... .......... ..........
AKU-2019-282   .......... .......... .......... .......... .......... .......... .......... .......... ..........
AKU-2019-408   .......... .......... .......... .......... .......... .......... .......... .......... ..........
AKU-2019-460   .......... .......... .......... .......... .......... .......... .......... .......... ..........
AKU-2020-51    .......... .......... .......... .......... .......... .......... .......... .......... ..........
AKU-2020-94    .......... .......... .......... .......... .......... .......... .......... .......... ..........

                      640        650        660        670        680        690        700        710        720
               ....|....| ....|....| ....|....| ....|....| ....|....| ....|....| ....|....| ....|....| ....|....|
L40389.1       AGATCCTTGC TAGGTAAGGA AATGAGAGAC AAGTTGGACA CCGACTTCGC TTACTTGTAC AGTGACTTGG ACAAGGGTTT CACCCCAATT
EU219981.1     .......... .......... .......... .......... .......... .......... .......... .......... ..........
AKU-2009-625   .......... .......... .......... .......... .......... .......... .......... .......... ..........
AKU-2019-90    .......... .......... .......... .......... .......... .......... .......... .......... ..........
AKU-2019-134   .......... .......... .......... .......... .......... .......... .......... .......... ..........
AKU-2019-282   .......... .......... .......... .......... .......... .......... .......... .......... ..........
AKU-2019-408   .......... .......... .......... .......... .......... .......... .......... .......... ..........
AKU-2019-460   .......... .......... .......... .......... .......... .......... .......... .......... ..........
AKU-2020-51    .......... .......... .......... .......... .......... .......... .......... .......... ..........
AKU-2020-94    .......... .......... .......... .......... .......... .......... .......... .......... ..........

                      730        740        750        760        770        780        790        800        810
               ....|....| ....|....| ....|....| ....|....| ....|....| ....|....| ....|....| ....|....| ....|....|
L40389.1       AACTTCGTCT TCCCTAACTT GCCTCTAGAA CACTACAGAA AGAGAGATCA TGCCCAACAA GCTATCTCTG GTACTTACAT GTCCTTGATT
EU219981.1     .......... .......... .......... .......... .......C.. .......... .......... .......... ..........
AKU-2009-625   .......... .......... .......... .......... .......C.. .......... .......... .......... ..........
AKU-2019-90    .......... .......... .......... .......... .......C.. .......... .......... .......... ..........
AKU-2019-134   .......... .......... .......... .......... .......C.. .......... .......... .......... ..........
AKU-2019-282   .......... .......... .......... .......... .......C.. .......... .......... .......... ..........
AKU-2019-408   .......... .......... .......... .......... .......C.. .......... .......... .......... ..........
AKU-2019-460   .......... .......... .......... .......... .......C.. .......... .......... .......... ..........
AKU-2020-51    .......... .......... .......... .......... .......C.. .......... .......... .......... ..........
AKU-2020-94    .......... .......... .......... .......... .......C.. .......... .......... .......... ..........

                      820        830        840        850        860        870        880        890        900
               ....|....| ....|....| ....|....| ....|....| ....|....| ....|....| ....|....| ....|....| ....|....|
L40389.1       AAGGAAAGAC GTGAGAAGAA CGATATCCAA AACCGTGACT TGATTGATGA ATTGATGAAG AACTCCACTT ACAAGGATGG TACTAAGATG
EU219981.1     .......... .......... ...C...... .......... .......... .......... .......... .......... ..........
AKU-2009-625   .......... .......... .......... .......... .......... .......... .......... .......... ..........
AKU-2019-90    .......... .......... ...C...... .......... .......... .......... .......... .......... ..........
AKU-2019-134   .......... .......... ...C...... .......... .......... .......... .......... .......... ..........
AKU-2019-282   .......... .......... .......... .......... .......... .......... .......... .......... ..........
AKU-2019-408   .......... .......... ...C...... .......... .......... .......... .......... .......... ..........
AKU-2019-460   .......... .......... .......... .......... .......... .......... .......... .......... ..........
AKU-2020-51    .......... .......... ...C...... .......... .......... .......... .......... .......... ..........
AKU-2020-94    .......... .......... .......... .......... .......... .......... .......... .......... ..........

                      910        920        930        940        950        960        970        980        990
               ....|....| ....|....| ....|....| ....|....| ....|....| ....|....| ....|....| ....|....| ....|....|
L40389.1       ACCGACCAAG AAATTGCCAA CCTATTGATT GGTGTCTTGA TGGGTGGTCA ACATACTTCC GCTGCTACCT CCGCTTGGTG TCTATTGCAT
EU219981.1     .......... .......... .......... .......... .......... .......... .......... .......... ..........
AKU-2009-625   .......... .......... .......... .......... .......... .......... .......... .......... ..........
AKU-2019-90    .......... .......... .......... .......... .......... .......... .......... .......... ..........
AKU-2019-134   .......... .......... .......... .......... .......... .......... .......... .......... ..........
AKU-2019-282   .......... .......... .......... .......... .......... .......... .......... .......... ..........
AKU-2019-408   .......... .......... .......... .......... .......... .......... .......... .......... ..........
AKU-2019-460   .......... .......... .......... .......... .......... .......... .......... .......... ..........
AKU-2020-51    .......... .......... .......... .......... .......... .......... .......... .......... ..........
AKU-2020-94    .......... .......... .......... .......... .......... .......... .......... .......... ..........
```

```
                    1000       1010       1020       1030       1040       1050       1060       1070       1080
            ....|....| ....|....| ....|....| ....|....| ....|....| ....|....| ....|....| ....|....| ....|....|
L40389.1     TTGGCTGAAA GACCAGATGT CCAAGAAGAA TTATACCAAG AACAAATGCG CGTCTTGAAC AACGATACCA AGGAATTGAC TTACGATGAC
EU219981.1   .......... .......... .......... ..G....... .......... .......... .......... .......... ..........
AKU-2009-625 .......... .......... .......... .......... .......... .......... .......... .......... ..........
AKU-2019-90  .......... .......... .......... ..G....... .......... .......... .......... .......... ..........
AKU-2019-134 .......... .......... .......... ..G....... .......... .......... .......... .......... ..........
AKU-2019-282 .......... .......... .......... .......... .......... .......... .......... .......... ..........
AKU-2019-408 .......... .......... .......... ..G....... .......... .......... .......... .......... ..........
AKU-2019-460 .......... .......... .......... .......... .......... .......... .......... .......... ...... ....
AKU-2020-51  .......... .......... .......... ..G....... .......... .......... .......... .......... ..........
AKU-2020-94  .......... .......... .......... ..G....... .......... .......... .......... .......... ..........

                    1090       1100       1110       1120       1130       1140       1150       1160       1170
            ....|....| ....|....| ....|....| ....|....| ....|....| ....|....| ....|....| ....|....| ....|....|
L40389.1     CTACAAAACA TGCCTCTATT GAACCAAATG ATCAAGGAAA CTTTGAGATT GCACCACCCA TTGCACTCTT TGTTCCGTAA AGTCATGAGA
EU219981.1   .......... .......... .......... .......... .......... .......... .......... .......... ..........
AKU-2009-625 .......... .......... .......... .......... .......... .......... .......... .......... ..........
AKU-2019-90  .......... .......... .......... .......... .......... .......... .......... .......... ..........
AKU-2019-134 .......... .......... .......... .......... .......... .......... .......... .......... ..........
AKU-2019-282 .......... .......... .......... .......... .......... .......... .......... .......... ..........
AKU-2019-408 .......... .......... .......... .......... .......... .......... .......... .......... ..........
AKU-2019-460 .......... .......... .......... .......... .......... .......... .......... .......... ..........
AKU-2020-51  .......... .......... .......... .......... .......... .......... .......... .......... ..........
AKU-2020-94  .......... .......... .......... .......... .......... .......... .......... .......... ..........

                    1180       1190       1200       1210       1220       1230       1240       1250       1260
            ....|....| ....|....| ....|....| ....|....| ....|....| ....|....| ....|....| ....|....| ....|....|
L40389.1     GATGTCGCTA TTCCAAACAC TTCCTACGTT GTCCCAAGGG ACTACCACGT TCTAGTCTCC CCAGGTTACA CTCACTTGCA AGAAGAATTC
EU219981.1   .......... .......... .......... .......... .......... .......... .......... .......... ..........
AKU-2009-625 .......... .......... .......... .......... .......... .......... .......... .......... ..........
AKU-2019-90  .......... .......... .......... .......... .......... .......... .......... .......... ..........
AKU-2019-134 .......... .......... .......... .......... .......... .......... .......... .......... ..........
AKU-2019-282 .......... .......... .......... .......... .......... .......... .......... .......... ..........
AKU-2019-408 .......... .......... .......... .......... .......... .......... .......... .......... ..........
AKU-2019-460 .......... .......... .......... .......... .......... .......... .......... .......... ..........
AKU-2020-51  .......... .......... .......... .......... .......... .......... .......... .......... ..........
AKU-2020-94  .......... .......... .......... .......... .......... .......... .......... .......... ..........

                    1270       1280       1290       1300       1310       1320       1330       1340       1350
            ....|....| ....|....| ....|....| ....|....| ....|....| ....|....| ....|....| ....|....| ....|....|
L40389.1     TTCCCTAAGC CAAATGAATT CAACATCCAC CGTTGGGACG GTGATGCTGC TTCTTCCAGT GCTGCTGGTG GTGACGAAGT TGATTACGGT
EU219981.1   .......... .......... .......... .......... .......... .......... .......... .......... ..........
AKU-2009-625 .......... .......... .......... .......... .......... .......... .......... .......... ..........
AKU-2019-90  .......... .......... .......... .......... .......... .......... .......... .......... ..........
AKU-2019-134 .......... .......... .......... .......... .......... .......... .......... .......... ..........
AKU-2019-282 .......... .......... .......... .......... .......... .......... .......... .......... ..........
AKU-2019-408 .......... .......... .......... .......... .......... .......... .......... .......... ..........
AKU-2019-460 .......... .......... .......... .......... .......... .......... .......... .......... ..........
AKU-2020-51  .......... .......... .......... .......... .......... .......... .......... .......... ..........
AKU-2020-94  .......... .......... .......... .......... .......... .......... .......... .......... ..........

                    1360       1370       1380       1390       1400       1410       1420       1430       1440
            ....|....| ....|....| ....|....| ....|....| ....|....| ....|....| ....|....| ....|....| ....|....|
L40389.1     TTCGGTGCTA TCTCCAAGGG TGTTTCCTCT CCATACTTGC CATTCGGTGG TGGTAGACAC AGATGTATCG GTGAATTGTT CGCTTACTGT
EU219981.1   .......... .......... .......... .......... .......... .......... .......... .......... ..........
AKU-2009-625 .......... .......... .......... .......... .......... .......... .......... .......... ..........
AKU-2019-90  .......... .......... .......... .......... .......... .......... .......... .......... ..........
AKU-2019-134 .......... .......... .......... .......... .......... .......... .......... .......... ..........
AKU-2019-282 .......... .......... .......... .......... .......... .......... .......... .......... ..........
AKU-2019-408 .......... .......... .......... .......... .......... .......... .......... .......... ..........
AKU-2019-460 .......... .......... .......... .......... .......... .......... .......... .......... ..........
AKU-2020-51  .......... .......... .......... .......... .......... .......... .......... .......... ..........
AKU-2020-94  .......... .......... .......... .......... .......... .......... .......... .......... ..........

                    1450       1460       1470       1480       1490       1500       1510       1520       1530
            ....|....| ....|....| ....|....| ....|....| ....|....| ....|....| ....|....| ....|....| ....|....|
L40389.1     CAATTGGGTG TGTTGATGTC CATTTTCATC AGAACCATGA AATGGCGTTA CCCAACTGAA GGTGAAACTG TCCCACCATC TGACTTCACC
EU219981.1   .......... .......... .......... .......... .......... .......... .......... .......... ..........
AKU-2009-625 .......... .......... .......... .......... .......... .......... .......... .......... ..........
AKU-2019-90  .......... .......... .......... .......... .......... .......... .......... .......... ..........
AKU-2019-134 .......... .......... .......... .......... .......... .......... .......... .......... ..........
AKU-2019-282 .......... .......... .......... .......... .......... .......... .......... .......... ..........
AKU-2019-408 .......... .......... .......... .......... .......... .......... .......... .......... ..........
AKU-2019-460 .......... .......... .......... .......... .......... .......... .......... .......... ..........
AKU-2020-51  .......... .......... .......... .......... .......... .......... .......... .......... ..........
AKU-2020-94  .......... .......... .......... .......... .......... .......... .......... .......... ..........
```

```
                    1540       1550       1560       1570       1580       1590       1600    1610       1620
              ....|....| ....|....| ....|....| ....|....| ....|....| ....|....| ....|....| ....|....| ....|....|
L40389.1      TCCATGGTCA CCCTACCAAC TGCCCCTGCT AAGATCTACT GGGAAAAGAG ACATCCAGAA CAAAAGTACT AGATGCTTTC AAAATATTAA
EU219981.1    .......... .......... ......A... .......... .......... .......... .......... .......... ..........
AKU-2009-625  .......... .......... .......... .......... .......... .......... .......... ..-------- ----------
AKU-2019-90   .......... .......... .......... .......... .......... .......... .......... ..-------- ----------
AKU-2019-134  .......... .......... ......A... .......... .......... G......... .......... ..-------- ----------
AKU-2019-282  .......... .......... .......... .......... .......... .......... .......... ..-------- ----------
AKU-2019-408  .......... .......... .......... .......... .......... .......... .......... ..-------- ----------
AKU-2019-460  .......... .......... ......A... .......... .......... G......... .......... ..-------- ----------
AKU-2020-51   .......... .......... .......... .......... .......... .......... .......... ..-------- ----------
AKU-2020-94   .......... .......... .......... .......... .......... .......... .......... ..-------- ----------

                    1630       1640       1650       1660       1670       1680       1690    1700       1710
              ....|....| ....|....| ....|....| ....|....| ....|....| ....|....| ....|....| ....|....| ....|....|
L40389.1      ACATTTTCTT CCAATAATCA ATGGTCAAAA CCTACTTCAA ACAATATTGC TGTCTGGCTC GTATACGGGA TATACGCTGA TTCATTAGCC
EU219981.1    .......... .......... .......... .......... .......... .......... .......... .......... ..........
AKU-2009-625  ---------- ---------- ---------- ---------- ---------- ---------- ---------- ---------- ----------
AKU-2019-90   ---------- ---------- ---------- ---------- ---------- ---------- ---------- ---------- ----------
AKU-2019-134  ---------- ---------- ---------- ---------- ---------- ---------- ---------- ---------- ----------
AKU-2019-282  ---------- ---------- ---------- ---------- ---------- ---------- ---------- ---------- ----------
AKU-2019-408  ---------- ---------- ---------- ---------- ---------- ---------- ---------- ---------- ----------
AKU-2019-460  ---------- ---------- ---------- ---------- ---------- ---------- ---------- ---------- ----------
AKU-2020-51   ---------- ---------- ---------- ---------- ---------- ---------- ---------- ---------- ----------
AKU-2020-94   ---------- ---------- ---------- ---------- ---------- ---------- ---------- ---------- ----------

                    1720       1730       1740       1750       1760       1770       1780    1790       1800
              ....|....| ....|....| ....|....| ....|....| ....|....| ....|....| ....|....| ....|....| ....|....|
L40389.1      TAATAGTAAG TCATCGTGAA TATCAACATG GATATTTCGG CATGACTTAA GCTGGTTGTT TCGTTTAGAG GGCTTTGCTG CTAAACGAAC
EU219981.1    .......... .......... .......... .......... .......... .......... .......... .......... ..........
AKU-2009-625  ---------- ---------- ---------- ---------- ---------- ---------- ---------- ---------- ----------
AKU-2019-90   ---------- ---------- ---------- ---------- ---------- ---------- ---------- ---------- ----------
AKU-2019-134  ---------- ---------- ---------- ---------- ---------- ---------- ---------- ---------- ----------
AKU-2019-282  ---------- ---------- ---------- ---------- ---------- ---------- ---------- ---------- ----------
AKU-2019-408  ---------- ---------- ---------- ---------- ---------- ---------- ---------- ---------- ----------
AKU-2019-460  ---------- ---------- ---------- ---------- ---------- ---------- ---------- ---------- ----------
AKU-2020-51   ---------- ---------- ---------- ---------- ---------- ---------- ---------- ---------- ----------
AKU-2020-94   ---------- ---------- ---------- ---------- ---------- ---------- ---------- ---------- ----------

                    1810       1820       1830       1840       1850       1860       1870    1880       1890
              ....|....| ....|....| ....|....| ....|....| ....|....| ....|....| ....|....| ....|....| ....|....|
L40389.1      TATACGATGT CTTCATCCGT ATAACTAGCA ATGTATGCAG TGCAAGCATA TGACAAAGTT GGTTGATCCC AAAGATTGTT TTAAAAAAAT
EU219981.1    .......... .......... .......... .......... .......... .......... .......... .......... ..........
AKU-2009-625  ---------- ---------- ---------- ---------- ---------- ---------- ---------- ---------- ----------
AKU-2019-90   ---------- ---------- ---------- ---------- ---------- ---------- ---------- ---------- ----------
AKU-2019-134  ---------- ---------- ---------- ---------- ---------- ---------- ---------- ---------- ----------
AKU-2019-282  ---------- ---------- ---------- ---------- ---------- ---------- ---------- ---------- ----------
AKU-2019-408  ---------- ---------- ---------- ---------- ---------- ---------- ---------- ---------- ----------
AKU-2019-460  ---------- ---------- ---------- ---------- ---------- ---------- ---------- ---------- ----------
AKU-2020-51   ---------- ---------- ---------- ---------- ---------- ---------- ---------- ---------- ----------
AKU-2020-94   ---------- ---------- ---------- ---------- ---------- ---------- ---------- ---------- ----------

                    1900       1910       1920       1930       1940       1950       1960       1970    1980
              ....|....| ....|....| ....|....| ....|....| ....|....| ....|....| ....|....| ....|....| ....|....|
L40389.1      TCATTTTCAT GATCAAAATT CAATGCTTCT TATACTACAT TTTTTTCATT TTCATTCATC TTTATTTTGG TGCCATTAAC ATATAGACTT
EU219981.1    .......... .......... .......... .......... .......... .......... .......... .......... ..........
AKU-2009-625  ---------- ---------- ---------- ---------- ---------- ---------- ---------- ---------- ----------
AKU-2019-90   ---------- ---------- ---------- ---------- ---------- ---------- ---------- ---------- ----------
AKU-2019-134  ---------- ---------- ---------- ---------- ---------- ---------- ---------- ---------- ----------
AKU-2019-282  ---------- ---------- ---------- ---------- ---------- ---------- ---------- ---------- ----------
AKU-2019-408  ---------- ---------- ---------- ---------- ---------- ---------- ---------- ---------- ----------
AKU-2019-460  ---------- ---------- ---------- ---------- ---------- ---------- ---------- ---------- ----------
AKU-2020-51   ---------- ---------- ---------- ---------- ---------- ---------- ---------- ---------- ----------
AKU-2020-94   ---------- ---------- ---------- ---------- ---------- ---------- ---------- ---------- ----------

                    1990       2000       2010       2020       2030       2040       2050    2060       2070
              ....|....| ....|....| ....|....| ....|....| ....|....| ....|....| ....|....| ....|....| ....|....|
L40389.1      AAGTTTCATT TACTATTTTA TATTTATTAT AAAATTTAAA CCGTGTATAC AATACAATAT TATTTAACAA ATCGAACTCT TGTATTATTA
EU219981.1    .......... .......... .......... .......... .......... .......... .......... .......... ..........
AKU-2009-625  ---------- ---------- ---------- ---------- ---------- ---------- ---------- ---------- ----------
AKU-2019-90   ---------- ---------- ---------- ---------- ---------- ---------- ---------- ---------- ----------
AKU-2019-134  ---------- ---------- ---------- ---------- ---------- ---------- ---------- ---------- ----------
AKU-2019-282  ---------- ---------- ---------- ---------- ---------- ---------- ---------- ---------- ----------
AKU-2019-408  ---------- ---------- ---------- ---------- ---------- ---------- ---------- ---------- ----------
AKU-2019-460  ---------- ---------- ---------- ---------- ---------- ---------- ---------- ---------- ----------
AKU-2020-51   ---------- ---------- ---------- ---------- ---------- ---------- ---------- ---------- ----------
AKU-2020-94   ---------- ---------- ---------- ---------- ---------- ---------- ---------- ---------- ----------
```

```
                   2080       2090       2100
              ....|....| ....|....| ....|....| ....|....
L40389.1      TCAAACTTTA CCTTGGTAAG CCTAAAGAAA CCACGTTTT 2109
EU219981.1    .......... .......... .......... ......... 2109
AKU-2009-625  ---------- ---------- ---------- --------- 1602
AKU-2019-90   ---------- ---------- ---------- --------- 1602
AKU-2019-134  ---------- ---------- ---------- --------- 1602
AKU-2019-282  ---------- ---------- ---------- --------- 1602
AKU-2019-408  ---------- ---------- ---------- --------- 1602
AKU-2019-460  ---------- ---------- ---------- --------- 1602
AKU-2020-51   ---------- ---------- ---------- --------- 1602
AKU-2020-94   ---------- ---------- ---------- --------- 1602
```

Supplementary Figure 1b: Coding fragment of *ERG11* gene of *N. glabratus* showing synonymous mutation. Accessions number L40389.1 and EU219981.1 were used as reference sequence, and sequence start (561 =1). Synonymous mutation at positions T300C, A1023G, T1557A and A1581G are shown in the four panels, respectively. "." represent similarity (no change of nucleotide). For alignment BioEdit software was used.
